# Chaperone-assisted expression and purification of the AML-associated Src-family kinase Fgr in *Escherichia coli*

**DOI:** 10.1016/j.jbc.2025.111099

**Published:** 2025-12-22

**Authors:** Giancarlo Gonzalez-Areizaga, Thomas E. Smithgall

**Affiliations:** Department of Microbiology and Molecular Genetics, University of Pittsburgh School of Medicine, Pittsburgh, Pennsylvania, USA

**Keywords:** Src-family kinase, Fgr, Hck, tyrosine kinases, SH3 domain, SH2 domain, GroEL, chaperones

## Abstract

Fgr, a member of the Src family of nonreceptor tyrosine kinases, is expressed in myeloid hematopoietic cells and is a validated drug target in acute myeloid leukemia. Like other Src family kinases, Fgr consists of an N-terminal unique domain, SH3 and SH2 regulatory domains, a catalytic kinase domain, and a C-terminal tail. Notably, Fgr is constitutively active when ectopically expressed in fibroblasts, with kinase activity uncoupled from SH3–SH2 domain regulation, a feature that distinguishes it from other family members. To support structural and biochemical studies, we developed a chaperone-assisted method to express and purify soluble, active Fgr in *Escherichia coli*. Fgr was coexpressed with C-terminal Src kinase to maintain C-terminal tail phosphorylation and with the protein-tyrosine phosphatase 1B catalytic domain to keep the activation loop dephosphorylated. While this strategy stabilizes soluble Src and Hck in *E. coli*, it was insufficient for Fgr. To enhance solubility, we added coexpression with the *E. coli* GroEL–GroES chaperone complex. This significantly improved Fgr solubility, although the kinase initially remained bound to GroEL–GroES. Washing column-bound GroEL–GroES–Fgr with ATP–MgCl_2_ partially dissociated the chaperone complex, enabling isolation of pure Fgr through subsequent size-exclusion and ion exchange chromatography. This method reliably produced highly pure, active recombinant Fgr, with consistent yields of 1 mg per 2 l of bacterial culture.

Src-family kinases (SFKs) are nonreceptor tyrosine kinases essential in the regulation of diverse cellular events, including cell proliferation, differentiation, and migration (1). All Src-family members share a common domain organization consisting of a myristoylated N-terminal unique region followed by the conserved Src-homology (SH) domains, SH3 and SH2. These regulatory domains are connected to a bilobed kinase domain *via* a linker that forms a polyproline type II helix. A regulatory tail extends from the C-terminal end of the kinase domain with a conserved tyrosine phosphorylation site essential for negative regulation. The tail tyrosine is phosphorylated by a specialized regulatory kinase known as the C-terminal Src kinase or Csk (2). Crystal structures have been reported for several near-full-length SFKs, which lack the unstructured N-terminal unique domain, including Src (3, 4), Hck (5, 6, 7), and Fgr (8). These structures show that the SH3 domain engages the polyproline type II helix formed by the SH2-kinase linker, whereas the tyrosine-phosphorylated C-terminal tail engages the SH2 domain, resulting in a closed overall conformation. Moreover, mutations or ligands that disrupt these SH3- and SH2-mediated interactions induce conformational “opening,” often leading to kinase activation (9). These findings strongly suggest that SFKs are negatively regulated by intramolecular interactions involving their SH3 and SH2 domains, and that phosphorylation of the negative regulatory tail by Csk is required for downregulation of kinase activity in cells. One exception to this general principle is Fgr, in which regulatory domain displacement does not affect kinase activity, even though the kinase is phosphorylated on the tail and adopts the closed conformation (10).

Hck, Lyn, and Fgr are among a subset of SFKs primarily expressed in myeloid cells and are associated with the development of acute myeloid leukemia (AML) (11). Expression levels of these myeloid SFKs correlate with poor AML patient outcomes, making them compelling targets for anti-AML drug development (12). Our previous work has shown that ATP-site inhibitors of Fgr have distinct allosteric effects on the overall conformation of the kinase, with the C-helix-out inhibitor A-419259 reinforcing the closed conformation (8). In contrast, type II inhibitor TL02-59 causes allosteric displacement of the SH3 and SH2 domains from the linker and C-terminal tail, respectively (8). These findings underscore the need for near-full-length recombinant forms of Fgr and other Src family members for structural and biochemical studies of inhibitor action, which often require relatively large (milligram) amounts of purified recombinant kinase proteins.

We recently developed a bacterial expression system for SFKs in near-full-length forms, which included the SH3, SH2, and kinase domains along with a tyrosine-phosphorylated C-terminal tail, and a His-6 purification tag replacing the N-terminal unique region. In the case of Hck, we coexpressed Csk to maintain tail phosphorylation and the catalytic subunit of protein-tyrosine phosphatase 1B (PTP1B) to keep the activation loop tyrosine in the dephosphorylated state. This approach yielded soluble Hck protein in milligrams per liter yields; subsequent X-ray crystallography of the bacterially expressed form of Hck revealed the closed conformation with the SH2 domain bound to the tyrosine phosphorylated tail and the SH3 bound to the SH2-kinase linker (7). Similar results were obtained with an analogous construct of Src in this system, yielding crystallization quality recombinant protein (unpublished data). Unlike Hck and Src, however, coexpression of Fgr with Csk and PTP1B did not result in significant amounts of soluble protein in *Escherichia coli*, despite sequence and structural conservation with Hck and Src.

To solve this problem, we developed a chaperone-assisted method for the expression and purification of Fgr in *E. coli*. Chaperone-assisted protein expression has been extensively explored in the past as an avenue for purification of otherwise unstable and insoluble proteins in *E. coli* (13). Here, we found that overexpression of the *E. coli* GroEL–GroES chaperone complex greatly improved Fgr solubility. However, this approach came at the cost of GroEL–GroES–Fgr complex coelution after the first purification step. Given that the GroEL–GroES complex uses the energy released from the hydrolysis of ATP to release client proteins (14), a common method for removing chaperones relies on the use of buffers with ATP–MgCl_2_ to promote client protein release. Indeed, washing the column-bound complex with ATP–MgCl_2_ released free Fgr from the GroEL–GroES complex. Subsequent size-exclusion chromatography (SEC) and ion exchange consistently yielded 1 mg of active kinase protein per 2 l of bacterial culture. Overall, this chaperone-assisted expression and purification protocol produces highly pure and active recombinant Fgr in *E. coli* and may be applicable to other multidomain kinase systems that are otherwise difficult to produce in bacteria.

## Results

### Coexpression of GroEL–GroES improves Fgr solubility at the cost of chaperone–kinase complex coelution

**Figure 1:**
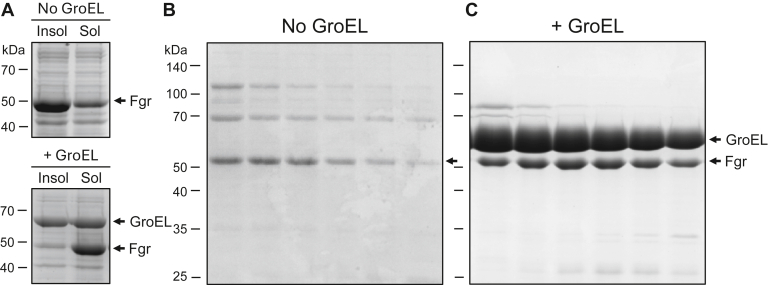
**Coexpression of *Escherichia coli* GroEL–GroES improves Fgr solubility.***A*, *B*acterial cultures expressing Fgr in the absence (*top panel*) or presence (*bottom panel*) of the GroEL–GroES complex were grown and induced as described under the *Experimental procedures* section. Aliquots were lysed, the soluble and insoluble fractions were separated by centrifugation, and the resulting protein fractions were separated by SDS-PAGE and stained with *Coomassie blue*. Soluble extracts were then subjected to Ni-IMAC chromatography. SDS polyacrylamide gels of the resulting peak fractions are shown for the samples without (*B*) or with (*C*) GroEL–GroES coexpression. Both gels are stained with *Coomassie blue*. Positions of the molecular weight markers are indicated on the *left* of all panels in kilodaltons. Ni-IMAC, nickel immobilized metal affinity chromatography.

Previous work has shown that coexpression of Hck or Src with Csk to maintain tail phosphorylation and PTP1B to keep the activation loop in the dephosphorylated state promotes expression of soluble kinase proteins in *E. coli* (7). In both expression constructs, the disordered N-terminal unique domain is replaced with a His-6 tag to promote purification by immobilized metal affinity chromatography (Ni-IMAC). However, coexpression of an analogous construct of Fgr with Csk and PTP1B in *E. coli* resulted in primarily insoluble Fgr protein following SDS-PAGE analysis of the cell lysate protein fractions by SDS-PAGE (Fig. 1*A*, *top panel*). Subsequent Ni-IMAC chromatography of the clarified lysate from 2 l of induced Fgr culture demonstrated elution peak fractions with very little recombinant Fgr (Fig. 1*B*). To improve solubility, we coexpressed Fgr with the chaperone complex, GroEL–GroES, in combination with the regulatory factors Csk and PTP1B as before. This approach greatly improved the solubility of Fgr as shown by SDS-PAGE analysis of the clarified cell extracts (Fig. 1*A*, *lower panel*) and in the subsequent Ni-IMAC peak fractions (Fig. 1*C*). This solubility improvement, however, came at the expense of GroEL–GroES–Fgr complex coelution, indicating that Fgr remained largely encapsulated within the GroEL–GroES complex. (For simplicity, we refer to the GroEL–GroES–Fgr complex as GroEL–Fgr below.)

### Washing the column-bound GroEL–Fgr complex with ATP releases the chaperone proteins

**Figure 2:**
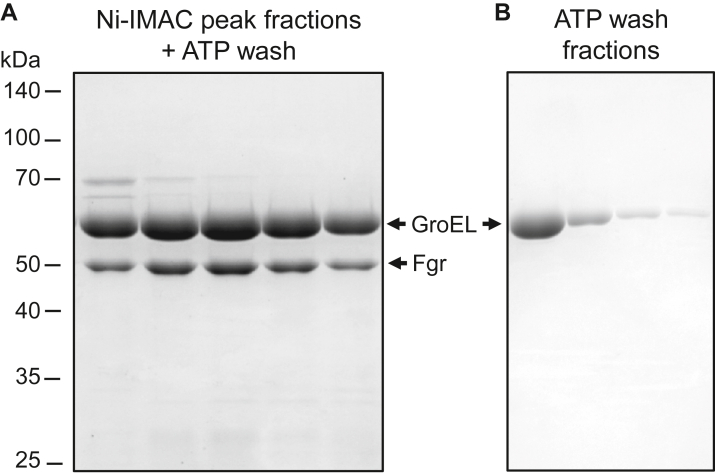
**Washing the column-bound GroEL-Fgr complex with ATP results in partial dissociation of GroEL.***A*, representative SDS-PAGE of the peak fractions from Ni-IMAC of Fgr coexpressed with GroEL–GroES after washing the column-bound Fgr–chaperone complex with ATP–MgCl_2_ buffer. Positions of the molecular weight markers are indicated on the *left* in kilodaltons. Note the increased ratio of GroEL to Fgr compared with the unwashed column fractions shown in Figure 1. *B*, fractions from the ATP–MgCl_2_ wash prior to elution. Ni-IMAC, nickel immobilized metal affinity chromatography.

Many chaperones, including the GroEL–GroES complex, make use of the energy of ATP hydrolysis to release folded substrate proteins from the complex, and addition of ATP–MgCl_2_ to purification buffers has been reported to enhance free recombinant proteins (14, 15). Therefore, we tested whether exposure of the GroEL–Fgr complex to high ATP concentrations would release Fgr from the complex. We found that washing the GroEL–Fgr complex with buffer containing 10 mM ATP while it was bound to the Ni-IMAC column partially dissociated GroEL–GroES from Fgr. This can be observed by the difference in the relative GroEL to Fgr protein band ratios following SDS-PAGE of the Ni-IMAC peak fractions with or without ATP washes (Fig. 2*A*; compare with (Fig. 1*B*). In addition, GroEL eluted from the column in the ATP wash (Fig. 2*B*), whereas recombinant Fgr remained column bound because of the N-terminal His6-tag. This result also implies that although Fgr is encapsulated by the GroEL–GroES complex to promote folding, the His6-tag remains exposed to the mobile phase to allow capture by the Ni-IMAC resin.

**Figure 3:**
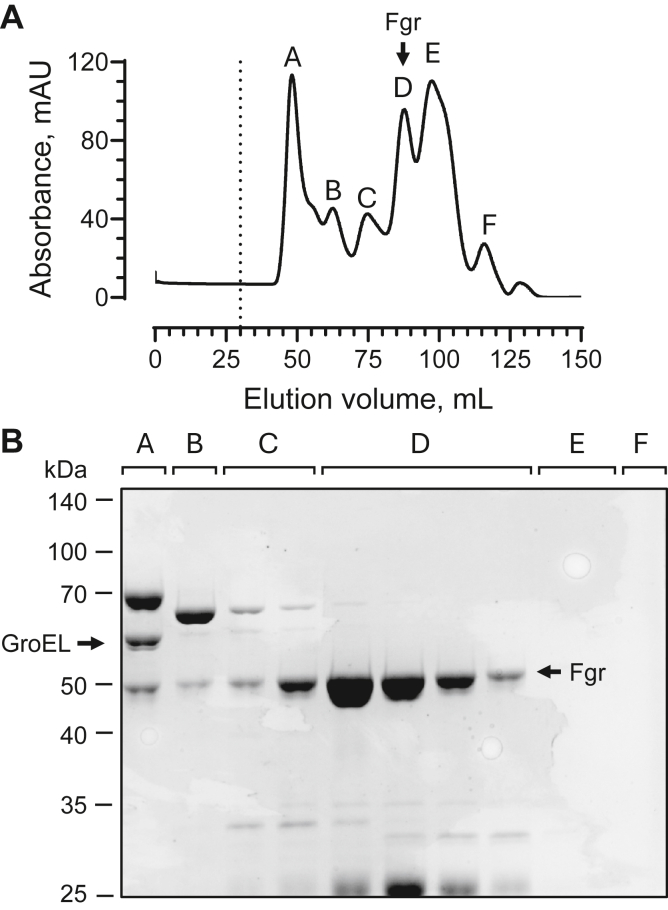
**Size-exclusion chromatography separates free Fgr from GroEL–GroES.***A*, representative Superdex 200 size-exclusion chromatogram following Ni-IMAC and ATP wash of the GroEL–Fgr complex. *B*, fractions from peaks identified by letters A–F in *A* were collected and analyzed by SDS-PAGE. Peak A, contained the remaining GroEL–Fgr complex, whereas peak D, contained the GroEL-free Fgr with some remaining contaminants. Peak E, while giving strong absorbance in the chromatogram, did not show a band on the gel. This peak may correspond to GroES, which has a subunit molecular weight of 10 kDa and therefore was not resolved. The positions of GroEL and Fgr are indicated by the *arrows*. Molecular weight markers are shown at *left*. Ni-IMAC, nickel immobilized metal affinity chromatography.

We then pooled the fractions containing Fgr following the ATP wash step and applied them to a preparative SEC column. This step separated the remaining GroEL–Fgr complex from unbound Fgr and other contaminants. A representative SEC elution profile and corresponding SDS-PAGE gel are shown in Figure 3, with Fgr present in peak D. In this chromatogram, an additional absorbance peak (peak E) was observed that did not result in a band on the gel. We suspect that this peak corresponds to GroES, which has a subunit molecular weight of 10 kDa and is therefore not resolved by 12% SDS-PAGE. Note that the SEC of pooled IMAC fractions containing the GroEL–Fgr complex that was not subjected to the ATP wash did not result in separation of the complex (data not shown), highlighting the importance of this step.

**Figure 4:**
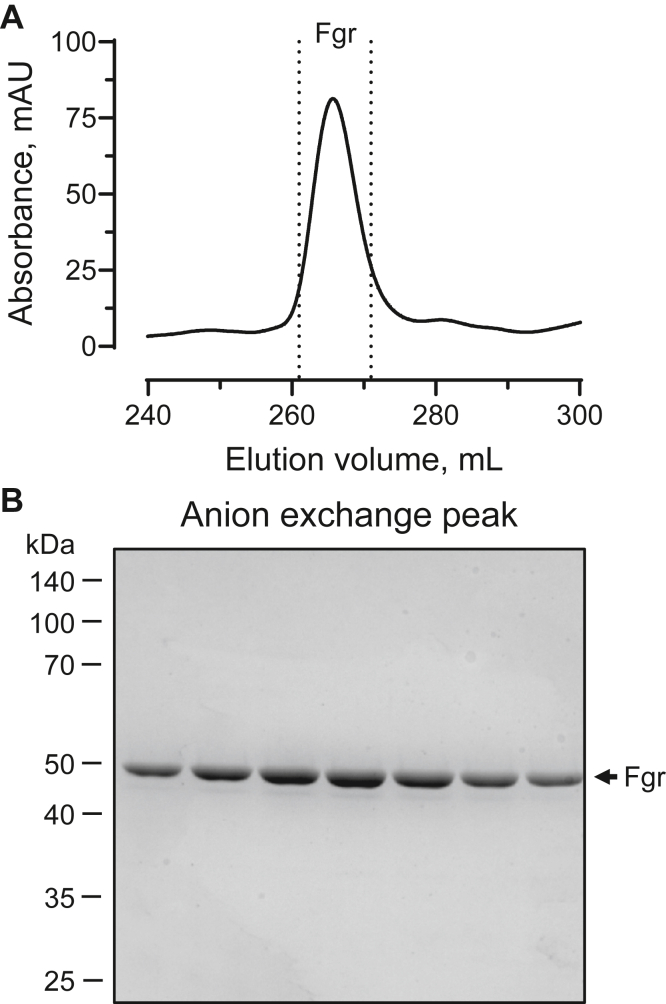
**Anion exchange chromatography of the SEC pool yields purified Fgr.***A*, SEC fractions enriched for Fgr were pooled and further purified on a HiTrap Q HP anion exchange column; a representative chromatogram is shown. Fractions from the main peak delineated by the *dotted lines* were analyzed by SDS-PAGE. *B*, representative SDS-PAGE of the peak fractions following anion exchange chromatography. These fractions were pooled, concentrated, and assayed for kinase activity. SEC, size-exclusion chromatography.

As a final purification step, we pooled the fractions containing “GroEL-free” Fgr and applied them to an anion exchange chromatography column. A single elution peak was observed in the chromatogram (Fig. 4*A*), which consisted of pure Fgr by SDS-PAGE (Fig. 4*B*). Four independent replicates of this method yielded an average of 1.04 ± 0.12 mg of pure Fgr per 2 l of starting culture.

### Chaperone-assisted purification of recombinant Fgr from *E. coli* yields a functional protein kinase

**Figure 5:**
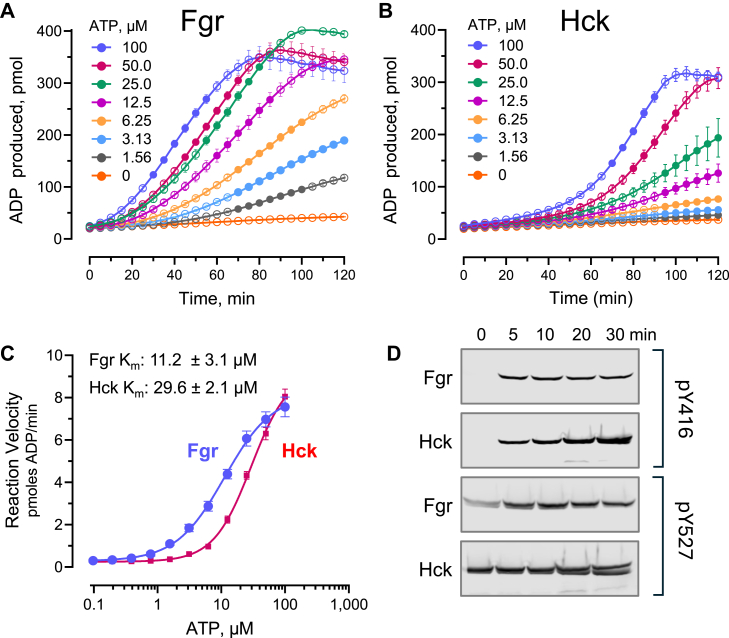
**Assessment of recombinant Fgr kinase activity.** Representative progress curves of ADP production by Fgr (*A*) and Hck (*B*) as a function of time with constant protein kinase and substrate concentrations and the ATP concentrations indicated. Hck (positive control) was also expressed in *Escherichia coli*, although the use of GroEL–GroES was not required. Each data point is the average of four technical replicates ± SD. *Solid circles* represent the linear portion of each curve that was fit by regression analysis to determine the steady-state reaction rate. At high concentrations of both kinases, the curves plateau and then slope downward as the peptide substrate is exhausted. *C*, representative concentration–response curve of the steady-state ADP production rate as a function of ATP concentration for near-full-length Fgr *versus* Hck. Average *K*_*m*_ values for ATP ± SD were determined from three independent experiments by nonlinear curve fitting using GraphPad Prism. *D*, time course of Fgr and Hck autophosphorylation. Purified Fgr and Hck autophosphorylation was followed over the time course shown by immunoblotting with phosphospecific antibodies to the activation loop (pY416) and tail (pY527) tyrosine phosphorylation sites. The experiment was run under conditions that closely mirror the kinetic kinase assays with respect to kinase concentration and with ATP set to the respective *K*_*m*_ values.

To ensure that the purified Fgr resulting from the chaperone-assisted purification protocol described above was functional, we measured the steady-state kinase activity using the ADP Quest assay. This assay reports the generation of ADP from ATP as a fluorescent signal in the presence of a peptide substrate as a function of time (16). As a positive control, we assayed near-full-length Hck that was expressed in the same *E. coli* system, although use of GroES–EL was not required (7). Both kinases were active in this assay, consistent with prior studies using equivalent SFK constructs expressed in bacteria as well as insect cells (7, 10). Representative reaction progress curves for Fgr and Hck over a range of ATP concentrations are shown in Figure 5, *A* and *B*, respectively. The linear portion of each curve was fit by linear regression analysis to determine the steady-state reaction velocity. These values were plotted *versus* the ATP concentrations to estimate the *K*_*m*_ values by direct nonlinear curve fitting (Fig. 5*C*).

The time required for Fgr to reach the steady state was much shorter than that observed for Hck, which may reflect the greater influence of the SH3 and SH2 domains on Hck kinase activity relative to Fgr (10, 17). To explore this possibility, we ran a time course of purified Fgr *versus* Hck autophosphorylation followed by immunoblotting with phosphospecific antibodies to the activation loop (pY416) and tail (pY527) tyrosine phosphorylation sites. The experiment was run under conditions that closely mirror the kinetic kinase assays with respect to kinase concentration and with ATP set to the respective *K*_*m*_ values for each kinase. In the absence of ATP, no band was observed with the activation loop pY416 antibody for either kinase, consistent with coexpression of each kinase with the PTP1B phosphatase (Fig. 5*D*). Both kinases bear pTyr on the tail as expected because of the presence of Csk. Interestingly, Fgr was maximally phosphorylated on the activation loop at the earliest time point sampled (5 min), whereas Hck underwent autophosphorylation more gradually over the incubation period. These results are consistent with the kinetic kinase assays, in which Fgr reaches steady state more rapidly than Hck.

Table 1 presents a comparison of the kinetics of recombinant Fgr and Hck proteins expressed in this bacterial system with previous results for the same constructs following expression in insect cells. For both Fgr and Hck, the apparent *K*_*m*_ value for ATP observed with the bacterially expressed proteins is about half that observed with kinases from insect cells. In terms of specific catalytic activity, bacterial Fgr was about twice that of the insect cell kinase. The specific activities of the two forms of Hck were similar, with the insect cell kinase about 20% higher than the bacterial form.

**Table 1:** Properties of recombinant near-full-length Fgr and Hck expressed in various hosts

Kinase	Host	ATP *K*_*m*_ (μM)	Specific activity (pmol ADP/min/μg)	Reference
Fgr	*Escherichia coli*	11.2 ± 3.1	22.8 ± 1.9	This study
Fgr	Sf9 insect cells	21.3^a^	9.3 ± 1.3	Shen *et al.* (10)
Hck	*Escherichia coli*	27.7 ± 18.5	32.2 ± 2.3	Selzer *et al.* (30)
Hck	Sf9 insect cells	55.8 ± 18.5	39.2 ± 2.8	Moroco *et al.* (17)

Recombinant kinases were assayed *in vitro* using the ADP Quest kinetic kinase assay except as noted.

a Value generated using the Z’Lyte kinase assay.

## Discussion

**Figure 6:**
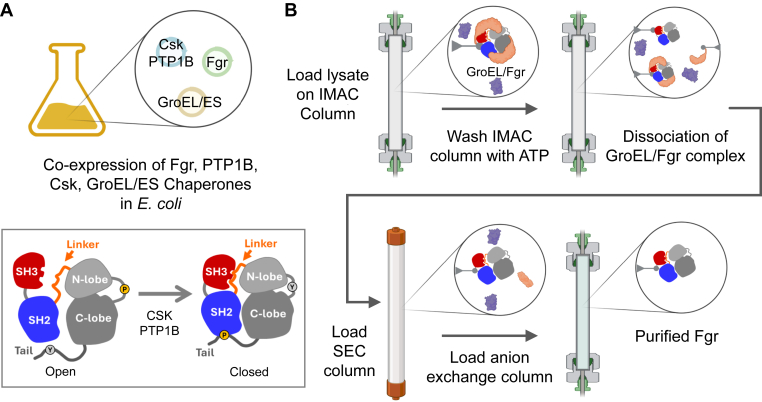
**Overview of chaperone-assisted expression and purification of Fgr in *Escherichia coli*.***A*, near-full-length Fgr (SH3–SH2–kinase–tail) was coexpressed in *E. coli* with the C-terminal Src kinase (Csk) and the protein-tyrosine phosphatase 1B (PTP1B) catalytic domain to promote interaction of the regulatory domains (SH3, *red*; SH2, *blue*) with the kinase domain (*gray*), resulting in a single, closed conformation (*inset*). Coexpression of the chaperone complex GroEL–GroES was used to improve protein solubility. *B*, purification strategy. Following induction of Fgr expression, the clarified cell lysate was loaded on a Ni-IMAC column, resulting in capture of the GroEL–GroES–Fgr complex. Dissociation of this complex was achieved by washing the Ni-IMAC column with ATP–MgCl_2_ buffer, resulting in partial release of GroEL–GroES from Fgr. Following elution, fractions with Fgr were pooled, concentrated, and applied to a Superdex 200 size-exclusion column, which resulted in the separation of Fgr from GroEL-GroES. These fractions were pooled and further purified by anion exchange chromatography, resulting in highly pure Fgr. Ni-IMAC, nickel immobilized metal affinity chromatography; SH, Src homology.

Here, we report an alternative protocol for the expression and purification of the near-full-length Fgr kinase in *E. coli*. In addition to the natural regulatory factors, Csk and PTP1B, successful expression of kinase-active Fgr required coexpression with the *E. coli* GroEL–GroES chaperone complex to promote Fgr folding and improve solubility. Although this approach resulted in the coelution of a GroEL–Fgr complex after the first purification step (Ni-IMAC affinity chromatography), we were able to promote chaperone–client protein release using an ATP buffer wash of the column-bound complex. While we attempted other chaperone-release procedures, including preincubation of sample lysates with ATP–MgCl_2_ prior to Ni-IMAC column loading or washing the column-bound complex with chaotropic agents, including urea and glucose (15), these methods did not improve soluble protein yields. Soluble Fgr was then purified by sequential SEC and anion exchange chromatography steps, consistently producing 1 mg of highly pure, active Fgr protein per 2 l of bacterial culture. An overview of the expression and purification protocol is illustrated in Figure 6.

Previous studies have employed coexpression of molecular chaperones to improve the soluble yield of difficult eukaryotic proteins in *E. coli*. Mammalian tyrosine kinases can be particularly challenging, because their hydrophobic catalytic domains frequently misfold and aggregate in the bacterial cytoplasm. Early studies demonstrated that GroEL–GroES overexpression enhanced the soluble recovery of other nonreceptor tyrosine kinases, including the SFKs, Fyn and Lck, as well as the SFK regulator, Csk (18, 19). Subsequent systematic analyses showed that GroEL–GroES, particularly when coexpressed with other chaperones, such as DnaK–DnaJ–GrpE, can significantly increase soluble yields of diverse recombinant kinases, including both tyrosine and Ser/Thr kinases (20, 21). However, the beneficial effects of GroEL–GroES are not universal. For example, Joseph *et al.* (22), reported that coexpression of GroEL–GroES did not improve solubility of the Itk tyrosine kinase domain, highlighting target-specific variability. Together, these observations show that while GroEL–GroES can facilitate soluble expression of many recombinant mammalian tyrosine kinases in *E. coli*, its effectiveness is influenced by the intrinsic folding properties of the individual kinases and therefore must be tested on a case-by-case basis.

In addition to GroEL–GroES, our chaperone coexpression system also included Skp, a small periplasmic folding factor also shown to enhance the solubility of heterologous proteins expressed in *E. coli*. Coexpression of Skp has been shown to prevent aggregation and increase the yield of functional single-chain antibody fragments (23, 24, 25, 26), whereas use of Skp as a solubility-enhancing fusion tag also improved folding of otherwise insoluble targets (27). Skp engages unfolded substrates through a dynamic, cage-like mechanism consistent with its ability to stabilize metastable intermediates during periplasmic folding in bacteria (28). While our study did not address the individual contribution of Skp to Fgr solubility, these previous studies suggest that Skp may also have supported Fgr folding in combination with GroEL–GroES.

## Experimental procedures

### Bacterial expression plasmids

The near-full-length Fgr coding sequence (human isoform, encoding amino acids 80–523) used for expression includes an N-terminal 6× His-tag followed by the SH3, SH2, and kinase domains and a modified C-terminal tail with the sequence YEEIP to enhance stable intramolecular interaction with the SH2 domain (10). This Fgr coding sequence was PCR amplified and subcloned into the pET-28a expression vector (Millipore Sigma). The complete nucleotide sequence of the Fgr coding region was confirmed prior to use. To facilitate expression of the near-full-length Fgr protein in the closed conformation, bacterial cells were cotransformed with a pET-DUET plasmid containing the PTP1B phosphatase catalytic domain (amino acids 1–283) and the full-length Csk kinase as described previously for Hck (7). To promote proper folding and solubility, cells were also transformed with the pACYC-Skp-GroEL–GroES expression plasmid encoding cytoplasmic copies of the Skp and GroEL–GroES chaperones (AddGene #83922) (29). One Shot BL21 (DE3) STAR chemically competent *E. coli* cells (Thermo Fisher Scientific) were transformed with all three expression plasmids using the provider’s instructions.

### Recombinant Fgr expression and purification

Bacterial cells were grown to an absorbance of 0.6 to 0.8 at 600 nm at 37 °C in 1 l of volume of terrific broth with 50 μg/ml carbenicillin, 30 μg/ml kanamycin, and 34 μg/ml chloramphenicol to select for each of the expression plasmids. The incubation temperature was then reduced to 16 °C, and protein expression was induced by the addition of IPTG to a final concentration of 0.25 mM overnight. Following induction, the cells were pelleted *via* centrifugation and either immediately lysed or snap frozen in liquid nitrogen and stored at −80 °C. Cells were suspended in Ni-IMAC binding buffer (25 mM Tris–HCl, pH 8.2, 500 mM NaCl, 20 mM imidazole, 2 mM β-mercaptoethanol [BME], and 10% glycerol) supplemented with cOmplete EDTA-free protease inhibitor cocktail (MilliporeSigma) prior to lysis *via* microfluidizer. The soluble protein fraction was isolated by ultracentrifugation at 100,000*g* for 1 h at 4 °C. The clarified lysates were then loaded onto a 5 ml HisTrap HP column (Cytiva). Bound proteins were then washed with 10 column volumes of Ni-IMAC binding buffer. The column was then washed with 40 column volumes of GroEL dissociation buffer (20 mM Hepes, pH 7.5, 10 mM MgCl_2_, 150 mM KCl, and 10 mM ATP). Bound proteins were eluted with a 0% to 100% gradient of Ni-IMAC elution buffer (25 mM Tris-HCl, pH 8.2, 500 mM NaCl, 500 mM imidazole, 2 mM BME, and 10% glycerol). Fractions containing Fgr were identified by SDS-PAGE, pooled, and dialyzed against SEC buffer (25 mM Tris–HCl, pH 8.3, 150 mM NaCl, 10% glycerol, and 2 mM BME) and run over a HiLoad 16/600 Superdex 200 pg preparative SEC column (Cytiva). For the final step, fractions containing “GroEL–GroES-free” Fgr were pooled and dialyzed against SEC buffer with 30 mM NaCl. The dialyzed Fgr protein was loaded onto a 5 ml HiTrap Q HP anion exchange column (Cytiva) followed by gradient elution with SEC buffer containing 1 M NaCl. The purity and integrity of the final Fgr protein were verified by SDS-PAGE.

### *In vitro* kinase assays

The activity of near-full-length Fgr and Hck was measured using the ADP Quest kinetic kinase assay (DiscoverX/Eurofins), which fluorometrically follows the production of ADP (16). Recombinant Hck used for these experiments was expressed in *E. coli* and purified as described (7). Kinase reactions (50 μl) were performed in low-volume black 384-well plates with a nonbinding surface (Corning; #3676) in kinase assay buffer (15 mM Hepes, pH 7.4, 20 mM NaCl, 1 mM EGTA, 0.02% Tween-20, 10 mM MgCl_2_, and 0.1 mg/ml bovine γ-globulins). To determine the apparent ATP *K*_*m*_, kinases were assayed at 150 ng/well and ATP was titrated by 1:1 serial dilutions starting at 100 μM with the substrate peptide (YIYGSFK) concentration held constant (500 μM). Fluorescence was measured every 5 min for 3 h using a SpectraMax i3x plate reader (Molecular Devices). The fluorescence values for each condition were averaged across four technical replicates. For each ATP concentration, the linear section of the progress curve was fit by regression analysis to estimate the steady-state reaction velocity (*V*_ss_). Nonlinear regression analysis of plots of *V*_ss_ *versus* ATP concentration yielded the apparent *K*_*m*_ values. Fluorescence values were converted to pmol ADP using a standard curve of known ADP concentrations. Statistical analysis was performed using GraphPad Prism (version 10; GraphPad Software, Inc).

To assess activation loop autophosphorylation, aliquots of recombinant Fgr and Hck were incubated at the same protein concentrations used for the kinetic assays and in the same kinase assay buffer. Reactions were initiated by the addition of ATP to a final concentration of 100 μM. Aliquots were removed at various time points, and the reactions were quenched by heating in SDS-PAGE sample buffer. Samples were resolved by SDS-PAGE, transferred to nitrocellulose, and immunoblotted with phosphospecific antibodies to the activation loop phosphotyrosine and the Csk-phosphorylated tail (pY416 and pY527, respectively; Thermo Fisher Scientific, catalog nos.: PA5-117390 and PA5-37592).

## Data availability

All data are described in the article.

## Conflict of interest

The authors declare that they have no conflicts of interest with the contents of this article.
